# The Early HOSPITAL Score to Predict 30-Day Readmission Soon After Hospitalization: a Prospective Multicenter Study

**DOI:** 10.1007/s11606-023-08538-0

**Published:** 2023-12-13

**Authors:** Philippe Mathys, Lukas Bütikofer, Daniel Genné, Jörg D. Leuppi, Marco Mancinetti, Gregor John, Drahomir Aujesky, Jacques D. Donzé

**Affiliations:** 1 Division of General Internal Medicine, Inselspital, Bern University Hospital, University of Bern, Bern, Switzerland; 2 Department of Medicine, Geneva University Hospitals, Geneva, Switzerland; 3 CTU Bern, University of Bern, Bern, Switzerland; 4 Division of Internal Medicine, Centre Hospitalier de Bienne, Bienne, Switzerland; 5 University Center of Internal Medicine, Cantonal Hospital Baselland and University of Basel, Liestal, Switzerland; 6 Department of General Internal Medicine, Fribourg Cantonal Hospital, Fribourg, Switzerland; 7 Faculty of Science and Medicine, University of Fribourg, Fribourg, Switzerland; 8 Department of Medicine, Neuchâtel Hospital Network, Neuchâtel, Switzerland; 9 University of Geneva, Geneva, Switzerland; 10 Division of General Medicine and Primary Care, Brigham and Women’s Hospital, Harvard Medical School, Boston, MA USA; 11 Division of General Internal Medicine, CHUV, Lausanne University, Lausanne, Switzerland

**Keywords:** hospital readmission, score, prediction model

## Abstract

**Background:**

The simplified HOSPITAL score is an easy-to-use prediction model to identify patients at high risk of 30-day readmission before hospital discharge. An earlier stratification of this risk would allow more preparation time for transitional care interventions.

**Objective:**

To assess whether the simplified HOSPITAL score would perform similarly by using hemoglobin and sodium level at the time of admission instead of discharge.

**Design:**

Prospective national multicentric cohort study.

**Participants:**

In total, 934 consecutively discharged medical inpatients from internal general services.

**Main Measures:**

We measured the composite of the first unplanned readmission or death within 30 days after discharge of index admission and compared the performance of the simplified score with lab at discharge (simplified HOSPITAL score) and lab at admission (early HOSPITAL score) according to their discriminatory power (Area Under the Receiver Operating characteristic Curve (AUROC)) and the Net Reclassification Improvement (NRI).

**Key Results:**

During the study period, a total of 3239 patients were screened and 934 included. In total, 122 (13.2%) of them had a 30-day unplanned readmission or death. The simplified and the early versions of the HOSPITAL score both showed very good accuracy (Brier score 0.11, 95%CI 0.10–0.13). Their AUROC were 0.66 (95%CI 0.60–0.71), and 0.66 (95%CI 0.61–0.71), respectively, without a statistical difference (*p* value 0.79). Compared with the model at discharge, the model with lab at admission showed improvement in classification based on the continuous NRI (0.28; 95%CI 0.08 to 0.48;* p* value 0.004).

**Conclusion:**

The early HOSPITAL score performs, at least similarly, in identifying patients at high risk for 30-day unplanned readmission and allows a readmission risk stratification early during the hospital stay. Therefore, this new version offers a timely preparation of transition care interventions to the patients who may benefit the most.

**Supplementary Information:**

The online version contains supplementary material available at 10.1007/s11606-023-08538-0.

## INTRODUCTION

Hospital readmissions after an initial hospitalization are common and are associated with a significant burden for the patients, their families, and the healthcare system.^1–3^ The transition of care from hospital to ambulatory setting is a high-risk period, especially for patients with higher comorbidity which has been associated with a higher risk of readmission.^4–6^ Furthermore, each admission is associated with a risk of new complications^7, 8^ and it is estimated that 30% of the readmissions are preventable und thus avoidable.^9^

To identify the patients at higher risk for readmission is challenging, as providers are not able to accurately evaluate the risk^10^ and very few prediction model tools have been validated. The HOSPITAL score showed good performances in predicting 30-day readmission, and a very good generalizability. It has been now validated in four continents, six countries, more than 136,000 patients in both retrospective^11, 12^ and prospective cohorts,^13^ and therefore currently is the best validated prediction model when compared to other scores^14^ and has outperformed the LACE score in two recent studies.^11, 13^ The original version of the HOSPITAL score includes seven readily available variables, and the simplified version six variables (number of procedures being left out), and both showed similar performances.^15^

Since the HOSPITAL score or its simplified version is calculated shortly before discharge, it may limit the time available to prepare the discharge. This timely constraint is mainly due to use of the last measured hemoglobin and sodium level before discharge, leaving only the variable length of stay unknown at time of admission. Therefore, we aimed to assess in a prospective multicenter cohort whether the simplified HOSPITAL score would perform similarly by using hemoglobin and sodium level at time of admission instead of discharge.

## METHODS

### Study Design and Participants

We conducted a prospective multicenter cohort study, which included all consecutive patient discharges from July 2017 to March 2018 in the medical services of four mid-sized hospitals in Switzerland. The participating centers were (1) Centre Hospitalier de Bienne, (2) Fribourg Cantonal Hospital, (3) Cantonal Hospital Baselland, Liestal, and (4) Neuchâtel Hospital Network. All are 100–120-bed services in a community teaching hospital.

All consecutive adult patients planning to be discharged alive from the internal general medicine wards were screened for eligibility. We included patients who were discharged home or to a nursing home and who stayed for at least 24 h at the hospital. In Switzerland, nursing homes are convalescent long-term care. Therefore, patients transferred to another acute-care hospital or to rehabilitation facilities were not included. We excluded patients from whom we could not obtain an informed consent (unable, not willing, or not obtained before discharge), were not living in the country, could not speak one of the two main national languages (French or Swiss German), or did not have a phone number to be reached at. Written informed consent was obtained from all the participants prior to their enrollment. The trial protocol was approved by the institutional review board of each site. The study was entirely funded by the Swiss National Science Foundation (government funding).

### Study Outcome

The primary outcome was the composite of the number of patients who had an unplanned readmission or who died within 30 days after hospital discharge. Unplanned readmission was defined as an urgent or emergent hospitalization (i.e., not scheduled more than 24 h in advance for investigation or treatment) to any division of any acute care hospital and that happened within 30 days after discharge from the index hospital discharge. Death was defined as all-cause mortality occurring within 30 days after discharge. As patients who died may have been readmitted if they had not died, we included both readmission and death as a composite outcome. The patients who were first readmitted and then died in the hospital counted as both readmissions and deaths.

A trained study nurse collected the outcomes at 30 days after hospital discharge by standardized phone interview. For patients who could not be reached after three attempts, information was collected through the patient relatives or the patients’ primary care physician. Finally, the electronic health system of the discharge hospital was screened for readmission or death. A lost to follow-up was defined as a patient with no information obtained from any of these four sources, or who withdrew consent during the study period.

### Predictor Variables

We collected the variables of the HOSPITAL score based on the same definition of the original study. We collected the Hemoglobin and Sodium levels (letters “H” and “S”) both at admission (i.e., within the first 24 h of admission) and at discharge (i.e., last lab value available before discharge). Because not all of the hospitals had a specific oncology division, the variable “discharge from an *O*ncology service” (letter “O”) was replaced by “active *O*ncologic diagnosis on admission or during hospitalization,” i.e., in the past 5 years, including metastatic and non-metastatic solid tumors and hematologic malignancies.^13, 15, 16^ The following procedures (letter “P”) were taken into account in the original version for the HOSPITAL score: coronary angiography, angioplasty, thromboaspiration, stenting, pacemaker implantation, transesophageal echocardiography, esogastroduodenoscopy, endoscopic retrograde cholangio-pancreatography, coloscopy, bronchoscopy, biopsy, thoracocentesis, lumbal puncture, paracentesis, chemotherapy, radiotherapy, continuous pressure ventilation, intubation, transfusion (blood or platelets), graft, dialysis, operation, suture, electro-neuromyography, joint aspiration, cystoscopy, bone marrow aspiration/biopsy, magnetic resonance imaging, computer tomography, angiography, positron emission tomography, scintigraphy.^1^ We collected the variables “*I*ndex admission *T*ype” (letter “I” and “T”) and “number of hospital *A*dmission(s) during the previous year” (letter “A”) as in the original study. We adapted the cutoff for the length of stay (letter “L”) to the median length of stay in Switzerland (8 days instead of 5 days for the USA), as previously done.^13, 16^ The simplified version of the HOSPITAL score includes six variables with the number of procedures being left out (Table 1). The three versions of the HOSPITAL score were compared: the original HOSPITAL score, the simplified HOSPITAL score, and the early HOSPITAL score (simplified score with lab at admission).

**Table 1:** Simplified and Early HOSPITAL Scores for 30-Day Unplanned Readmissions and Death

Attribute	Points
Low **H**emoglobin level (< 12 g/dl) at admission/discharge^a^	1
Active **O**ncological disease	2
Low **S**odium level (< 135 mmol/l) at admission/discharge^a^	1
**I**ndex admission **T**ype: nonelective	1
Number of hospital **A**dmission(s) during the previous year:	
0–1	0
2–5	2
> 5	5
**L**ength of stay ≥ 8 days^b^	2

The patients were categorized into two groups according to the number of points (optimal cut-point: 4 points): unlikely (< 4 points) and likely (≥ 4 points) to be readmitted

^a^Variables used at admission or at discharge

^b^Adapted and validated in Switzerland^13, 15^

### Statistical Analysis

Baseline characteristics are presented using absolute and relative frequencies for categorical and median, lower quartile (lq), and upper quartile (uq) for continuous variables.

The area under the receiver operating characteristic (ROC) curve (AUROC) is presented with asymptotic normal 95% CI. The Brier score was calculated using internally predicted (estimated) probabilities and is presented with bias-corrected bootstrap 95% CI. Goodness of fit was assessed by comparing observed and predicted probabilities by deciles of the predicted probabilities. The three versions of the HOSPITAL score were categorized into two groups: unlikely and likely to be readmitted, according to the number of points. Optimal cut-points were 5 points for the HOSPITAL score, and 4 points for the simplified HOSPITAL score and the early HOSPITAL score (with lab values from admission). These were calculated according to the methods of Liu^17^ and Youden.^18^ At these cut-points, we calculated sensitivity (SE), specificity (SP), and predictive values (PV) with exact binomial 95%CI, likelihood ratios with the Katz 95%CI, and the diagnostic odds ratio with Cornfield 95%CI.

The original and simplified HOSPITAL scores were compared using the AUC (based on a non-parametric procedure suggested by DeLong et al.),^19^ and the category-free (or continuous) version of the net reclassification improvement or index (NRI)^20, 21^ with 95% bias-corrected bootstrap CI.

Readmission rates are presented with Wilson score 95% confidence intervals (CI). The composites of unplanned readmission and death were analyzed using the Kaplan–Meier estimator with 95%CI based on pointwise Greenwood standard errors. The competing risks for readmission and death (without readmission) were analyzed using the cumulative incidence with 95%CI.^22^

## RESULTS

During the study period, we screened a total of 3239 patients. Among them, 2305 (71.2%) were excluded: 653 (20.2%) because of not meeting inclusion criteria and 1652 (51%) because of the presence of exclusion criteria. We therefore enrolled 934 medical patients (Fig. S1). For five patients, the 30 days interview could not be done: four because of withdrawal of consent and one because of violation of inclusion criteria (not discharged home/nursing home). In total, 924 of the remaining 929 patients had the primary outcome available while five patients had missing data (one lost to follow-up, two withdrew consent, two incomplete data). The variables of the HOSPITAL scores and the baseline characteristics for participants with and without 30-day readmission or death are reported in Tables 2 and 3 (also Table S2). Among the included patients, 122 (13.2%) had a 30-day unplanned readmission or death. Of those, 111 (91%) were readmitted and 21 (17%) died. Of those readmitted, 10 were first readmitted and then died in the hospital. The median hemoglobin levels at admission and discharge were 13.2 and 12.4 g/dl, and the median sodium level was 137 and 139 mmol/l, respectively. The proportions of patients categorized as high risk were 28% for the original score and 34% for the two simplified score versions (258, 311, and 312 patients, respectively).

**Table 2:** Baseline Characteristics for Patients With and Without 30-Day Readmission or Death^a^

	Entire cohort (*N* = 934)		With 30-day readmission or death (*N* = 122)		Without 30-day readmission or death (*N* = 802)	
	Non-missing	Median [lq, uq] or *n* (%)	Non-missing	Median [lq, uq] or *n* (%)	Non-missing	Median [lq, uq] or *n* (%)
Age [years]	934	71.0 [58.0, 80.0]	122	73 [64, 82]	802	70 [57, 80]
Gender	934		122		802	
Male		526 (56%)		67 (55%)		454 (57%)
Female		408 (44%)		55 (45%)		348 (43%)
Hemoglobin level at discharge [g/dl]	919	12.4 [10.9, 13.7]	120	117 [99, 133]	790	125 [110, 138]
Hemoglobin level at discharge < 120 g/dl	934	377 (40%)	122	68 (56%)	802	303 (38%)
Hemoglobin level at admission [g/dl]	904	13.2 [11.7, 14.6]	120	123 [104, 140]	775	133 [118, 147]
Hemoglobin level at admission < 120 g/dl	934	257 (28%)	122	52 (43%)	802	203 (25%)
Diagnosis of active cancer	934	180 (19%)	122	47 (39%)	802	132 (16%)
Sodium level at discharge [mmol/l]	911	139 [137, 141]	120	139 [136, 140]	782	139 [137, 141]
Sodium level at discharge < 135 mmol/l	934	72 (7.7%)	122	13 (11%)	802	58 (7.2%)
Sodium level at admission [mmol/l]	900	137 [134, 139]	120	136 [133, 139]	771	137 [135, 139]
Sodium level at admission < 135 mmol/l	934	234 (25%)	122	38 (31%)	802	190 (24%)
Procedure during the hospitalization (any ICD-10 coded)	934	564 (60%)	122	82 (67%)	802	479 (60%)
Non-elective admission	934	859 (92%)	122	115 (94%)	802	736 (92%)
Number of hospitalizations at the same hospital in the last 12 months	934		122		802	
0–1		779 (83%)		95 (78%)		676 (84%)
2–5		150 (16%)		27 (22%)		121 (15%)
> 5		5 (0.5%)		0 (0%)		5 (0.6%)
Length of stay of current hospitalization [days]	934	6.00 [4.00, 9.00]	122	7.0 [5.0, 11]	802	6.0 [4.0, 9.0]
Length of stay ≥ 8 days	934	321 (34%)	122	55 (45%)	802	262 (33%)
Original HOSPITAL score	934	3.00 [2.00, 5.00]	122	4.5 [3.0, 6.0]	802	3.0 [2.0, 4.0]
Simplified HOSPITAL score	934	3.00 [1.00, 4.00]	122	4.0 [2.0, 5.0]	802	2.0 [1.0, 4.0]
Simplified HOSPITAL score with lab at admission	934	3.00 [1.00, 4.00]	122	4.0 [2.0, 5.0]	802	2.0 [1.0, 4.0]

^a^For 10 patients it was not known whether they were readmitted or died

**Table 3:** Performance of the Original HOSPITAL Score, the Simplified HOSPITAL Score, and the Early HOSPITAL Score (*N* = 924)

	Original HOSPITAL score	Simplified HOSPITAL score	Early HOSPITAL score
Area under the ROC	0.66 (0.61–0.71)	0.66 (0.60–0.71)	0.66 (0.61–0.71)
Optimal cut-point	≥ 5	≥ 4	≥ 4
Sensitivity (95% CI)	50% (41 to 59%)	57% (48 to 66%)	58% (49 to 67%)
Specificity (95% CI)	75% (72 to 78%)	70% (67 to 73%)	70% (67 to 73%)
Positive predictive value (95% CI)	24% (19 to 29%)	23% (18 to 28%)	23% (18 to 28%)
Negative predictive value (95% CI)	91% (88 to 93%)	92% (89 to 94%)	92% (89 to 94%)
Positive likelihood ratio (95% CI)	2.0 (1.6 to 2.5)	1.9 (1.6 to 2.3)	1.9 (1.6 to 2.3)
Negative likelihood ratio (95% CI)	0.66 (0.55 to 0.79)	0.61 (0.49 to 0.75)	0.60 (0.48 to 0.74)
Diagnostic odds ratio (95% CI)	3.1 (2.1 to 4.5)	3.1 (2.1 to 4.6)	3.2 (2.2 to 4.8)

The discriminatory power of the three score versions was almost identical with an AUROC of 0.66 (95%CI 0.61–0.71) for the original and early HOSPITAL score, and 0.66 (95%CI 0.60–0.70) for the simplified version. The overall comparison between the different versions of the score was not statistically significant (*p* value 0.96).

The sensitivity of the HOSPITAL score was slightly lower in comparison to the simplified score and its early version using the lab at admission (50% vs 57% and 58% respectively), while its specificity was slightly higher compared to both simplified scores (75% vs 70% and 70% respectively). The global performance of each score was similar in terms of positive and negative predictive values, as well as for the positive likelihood ratio (LR). The negative LR was slightly worse in the HOSPITAL score compared to both the simplified score and its early version (0.66, 0.61, and 0.60 respectively).

The Brier score of 0.11 (95%CI 0.10 to 0.13) indicated a good overall performance of the original HOSPITAL score. Both the simplified version of the HOSPITAL score and the early HOSPITAL score had the same accurate performance with a Brier score of 0.11 (95%CI 0.10 to 0.13).

**Figure 1:**
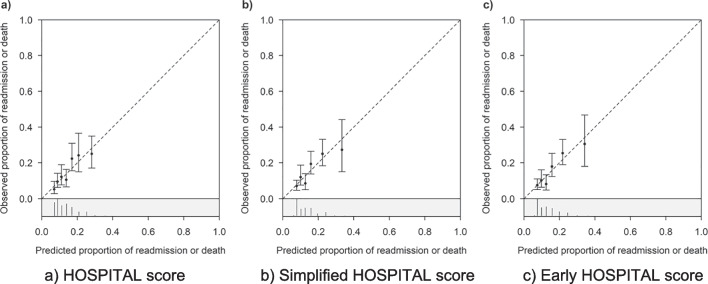
Goodness-of-fit for the three versions of the HOSPITAL score. Observed probabilities of readmission or death with 95% confidence intervals per decile of the internally predicted probabilities. The distribution of patients with (red) and without (black) readmissions or deaths is shown in the gray area.

All three scores showed a reasonable goodness-of-fit with *p* values from the Hosmer–Lemeshow test of 0.35, 0.34, and 0.22 for the original, early, and simplified HOSPITAL scores respectively (Fig. 1).

The net reclassification improvement (NRI) in the simplified compared to the original HOSPITAL score was as follows: − 0.15 (95%CI − 0.32 to 0.03) with a *p* value of 0.12. Compared with the model at discharge, the model with lab at admission (early version) showed improvement in classification based on the continuous NRI 0.28 (95%CI 0.08 to 0.48) with a significative *p* value of 0.004 mainly driven by an improvement in prediction of non-cases that compensated the worse prediction for cases: 75% (599/802) non-cases had a lower risk if lab at admission was used, while 39% (48/122) cases had a higher risk if lab at admission was used.

## DISCUSSION

This study validates the use of the HOSPITAL score using lab at admission instead of discharge, which allows a more timely decision-making. Not only the performance was identical with a C-statistic of 0.66, but we found an even better prediction of non-cases. Therefore, the early HOSPITAL score offers an interesting alternative to identify the risk of readmission earlier during the hospital stay. To our knowledge, the early HOSPITAL score is the only validated score that can be used early after admission to assess the risk of 30-day readmission and death.

Among the six variables included in the early HOSPITAL score, five are known at admission, and only the length of stay (LOS) above the median (≥ 8 days) would remain unknown until the threshold is reached. We showed in this study that only 15% of the patients would be reclassified from low risk to high risk once the length of stay exceeds the cut-off. Therefore, 85% of the patients may have their prediction calculated already at time of hospital admission. Other known readmission prediction models include variables that are available only at discharge or after discharge.

We can hypothesize that the similar performance using lab values at admission may be explained by quite similar values of hemoglobin and sodium between admission and discharge, which does not affect the score. These lab values probably reflect more the severity of the patient’s comorbidities than the state of health at a specific time (admission vs discharge). For example, it is expected that a patient with a hemoglobin level at admission lower than 12 g/dl will still leave the hospital with a hemoglobin below 12 g/dl, since the transfusion threshold is much lower. Regarding the low level of sodium, we identified more patients with hyponatremia at admission than at discharge. This was to be expected, but the purpose of the study was to show that by using the admission values, the score is still able to identify patients at high risk of admission. And indeed, the results showed that this is the case.

An early identification of high-risk patients may be indeed valuable. In a recent meta-analysis of 47 randomized controlled trials, it was shown in the subgroup analysis that interventions starting already during hospital stay and continuing after discharge were more effective in reducing readmissions compared to interventions starting after discharge.^23^ The study emphasizes that interventions enhancing patient empowerment were the most effective, and thus highlights the importance of patient’s education in reducing hospital readmissions. Therefore, the use of the early HOSPITAL score may offer to target patients at high risk of readmission earlier during the admission in order to anticipate the discharge and simplify the preparation, the organization of an intervention, and patient’s empowerment.

The study has to be interpreted considering some limitations. First, the findings might not apply to other patient populations, as any validation study. However, our study included patients discharged from the general internal medicine ward from different teaching hospital sizes, which included medical patients with multiple chronic conditions. Second, the only remaining variable not available at admission is the LOS. However, as mentioned above, only 15% of high-risk patients would have their risk classification changing due to the LOS, and most of these patients may have a predictable short versus long LOS at admission. Therefore, a patient who is high risk based on admission variables will remain at high risk regardless of length of stay, and may therefore be targeted for readmission reduction strategies early on. The strengths of this large study are the prospective and multicenter design, giving a high level of validation.

## CONCLUSION

The early HOSPITAL score showed overall good and similar performance in identifying patients at high risk for 30-day unplanned readmission. Interestingly, the early version using most of the parameters available already at admission showed similar good performance and could be useful in identifying patients earlier before their hospital discharge. Therefore, this early HOSPITAL score version offers a readmission risk stratification early during the hospital stay, which allows a timely preparation of transition care interventions to the patients who may benefit the most. Using the early HOSPITAL score might allow to target high-risk patients, who will more benefit from an intervention and thus reduce readmission.

### Supplementary Information

Below is the link to the electronic supplementary material.

Supplementary file1 (DOCX 107 kb)
