# Supplementary material for: The Early HOSPITAL Score to Predict 30-Day Readmission Soon After Hospitalization: a Prospective Multicenter Study
Source: J Gen Intern Med. 2023 Dec 13;39(5):756–61. doi: 10.1007/s11606-023-08538-0 (PMC11043245; doi:10.1007/s11606-023-08538-0)
Supplement: Supplementary file 1 — Supplementary file1 (DOCX 107 kb) [file 11606_2023_8538_MOESM1_ESM.docx]

**Appendix:**

**Figure S1.** Study Flow Chart.

**
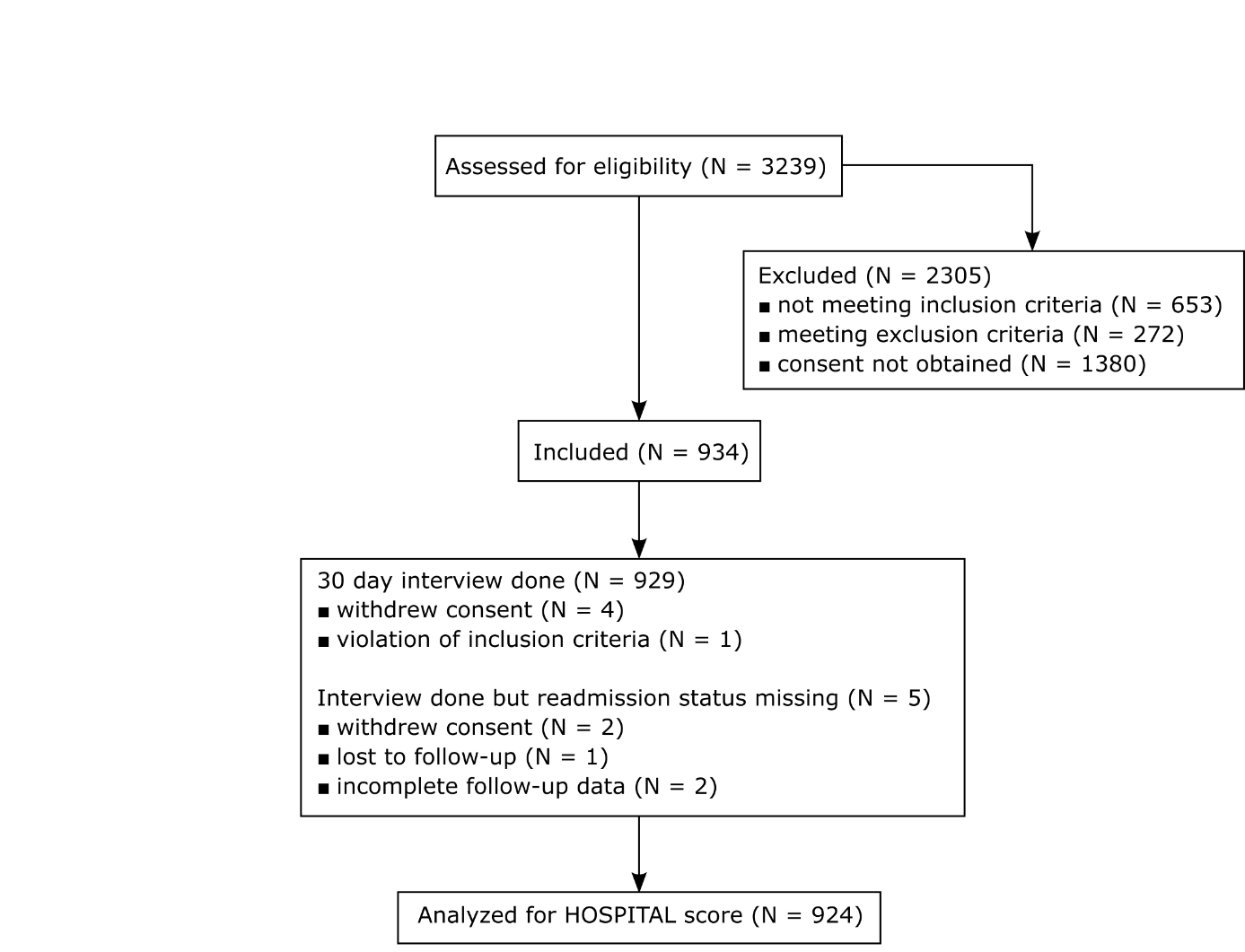
**

**Table S2**

Proportion and readmission rate per score points for the 3 versions of the HOSPITAL score.

| **HOSPITAL score** | **Original score** | | | **Simplified score with lab at discharge** | | | | **Simplified score with lab at admission** | | | |
| --- | --- | --- | --- | --- | --- | --- | --- | --- | --- | --- | --- |
|  | **N** | **Readmission or deaths** | **Readmission rate (95% CI)** | **N** | **Readmission or deaths** | **Readmission rate (95% CI)** | **N** | | **Readmission or deaths** | **Readmission rate (95% CI)** |  |
| **0** | 3 | 0 | 0.0% (0.0 to 56%) | 17 | 0 | 0.0% (0.0 to 18%) | 18 | | 0 | 0.0% (0.0 to 18%) |  |
| **1** | 169 | 9 | 5.3% (2.8 to 10%) | 310 | 23 | 7.4% (5.0 to 11%) | 287 | | 23 | 8.0% (5.4 to 12%) |  |
| **2** | 211 | 20 | 9.5% (6.2 to 14%) | 133 | 16 | 12% (7.5 to 19%) | 147 | | 15 | 10% (6.3 to 16%) |  |
| **3** | 131 | 16 | 12% (7.7 to 19%) | 153 | 13 | 8.5% (5.0 to 14%) | 160 | | 13 | 8.1% (4.8 to 13%) |  |
| **4** | 152 | 16 | 11% (6.6 to 16%) | 150 | 29 | 19% (14 to 26%) | 134 | | 24 | 18% (12 to 25%) |  |
| **5** | 112 | 25 | 22% (16 to 31%) | 51 | 15 | 29% (19 to 43%) | 79 | | 21 | 27% (18 to 37%) |  |
| **6** | 58 | 14 | 24% (15 to 37%) | 77 | 17 | 22% (14 to 33%) | 63 | | 15 | 24% (15 to 36%) |  |
| **7** | 58 | 14 | 24% (15 to 37%) | 18 | 5 | 28% (12 to 51%) | 19 | | 8 | 42% (23 to 64%) |  |
| **8** | 16 | 4 | 25% (10 to 49%) | 11 | 4 | 36% (15 to 65%) | 12 | | 3 | 25% (8.9 to 53%) |  |
| **9** | 10 | 4 | 40% (17 to 69%) | 2 | 0 | 0.0% (0.0 to 66%) | 3 | | 0 | 0.0% (0.0 to 56%) |  |
| **10** | 2 | 0 | 0.0% (0.0 to 66%) | 0 | 0 | n.d. | 1 | | 0 | 0.0% (0.0 to 79%) |  |
| **11** | 1 | 0 | 0.0% (0.0 to 79%) | 2 | 0 | 0.0% (0.0 to 66%) | 1 | | 0 | 0.0% (0.0 to 79%) |  |
| **12** | 1 | 0 | 0.0% (0.0 to 79%) | 0 | 0 | n.d. | 0 | | 0 | n.d. |  |
| **13** | 0 | 0 | n.d. | / | / | / | / | | / | / |  |
| **Overall** | 924 | 122 | 13% (11 to 16%) | 924 | 122 | 13% (11 to 16%) | 924 | | 122 | 13% (11 to 16%) |  |
